# Proposal for a New Questionnaire for the Early Identification of Patients With Obstructive Sleep Apnea Syndrome

**DOI:** 10.7759/cureus.97675

**Published:** 2025-11-24

**Authors:** Bruno Bordoni, Marta Simonelli, Bruno Morabito, Allan R Escher

**Affiliations:** 1 Physical Medicine and Rehabilitation, Foundation Don Carlo Gnocchi, Milan, ITA; 2 Rehabilitation Medicine, Ospedale dei Castelli, Azienda Sanitaria Locale (ASL) Rome, Rome, ITA; 3 Physical Medicine and Rehabilitation, School of Osteopathic Centre for Research and Studies, Milan, ITA; 4 Oncologic Sciences, University of South Florida Morsani College of Medicine, Tampa, USA; 5 Anesthesiology and Pain Medicine, H. Lee Moffitt Cancer Center and Research Institute, Tampa, USA

**Keywords:** continuous positive airways pressure, cpap, diaphragm, obstructive sleep apnea syndrome, osas, osteopathic manipulation, physiotherapy, rehabilitation, tongue

## Abstract

Obstructive sleep apnea syndrome (OSAS) is a very common, but underestimated, disorder. OSAS is characterized by repeated apneas during sleep due to upper airway obstruction. This condition can cause the onset of several chronic diseases, with increased mortality and morbidity. The test of choice to verify the presence of OSAS is polysomnography, a tool considered the gold standard. Not all questionnaires include the comorbidities of the patient with OSAS, which are also found in high percentages, and which can confuse the main problem of apneas. The aim of this article is to present a new questionnaire that intercepts in advance the patient with OSAS, considering different comorbidity variables. The proposed evaluation tries to make the best use of the information collected from the literature, framing the patient's clinical situation with more information. Further studies will be needed to validate the innovative approach proposed in the article.

## Introduction

Obstructive sleep apnea syndrome (OSAS) is characterized by repeated apneas during the sleep cycle due to upper airway obstruction, caused by inadequate function and performance of the pharyngeal dilator muscle complex with respect to the intraluminal negative pressure forces resulting from inspiratory muscle contraction [1,2]. Recent epidemiological data from 2019 indicate that 936 million adults (36-69 years) in the world are diagnosed as patients with severe OSAS, and 425 million adults (30-69 years) are identified as patients with moderate to severe OSAS, with peaks in countries with a larger population (China, the United States, Brazil, India). Compared to the 2007 data from the World Health Organization (WHO), which estimated that around 100 million people were affected by OSAS, there is a significant disparity with the most recent data, likely due to a more rigorous statistical approach used in 2019 [3].

The criteria for a diagnosis of OSAS were established in 1999 by the American Academy of Sleep Medicine Task Force, also known as the “Chicago criteria” [4]. The criteria specify that a patient with OSAS must have sleep-disordered breathing (SDB), previously measured with instruments (polysomnography, the gold standard) during hours of rest, and possible episodes of sleepiness during the day - excessive daytime sleepiness (EDS) [4]. The diagnosis is made based on the apnea-hypopnea index (AHI) values per hour. With an AHI between 15 and 30, the disorder is considered moderate; an AHI over 30 indicates a severe disorder (with evident clinical signs); a mild disorder falls with an AHI between 5-15 [4]. This definition also falls within the International Classification of Sleep Disorders (2021, ICSD-3).

AHI values increase with age, while lower values should be found in younger patients; yet, contrary to numerical logic, mortality and some cardiovascular comorbidities increase in younger patients [5]. Hypopnea is defined as: “a drop of ≥30% in breathing amplitude and in oxygen saturation >3% (by the American Academy of Sleep Medicine, AASM), or >4% (by the Center for Medicare and Medicaid Services, CMMS)” [6]. The apneic event is defined as: “the absence or greater than 90% reduction of inspiratory airflow for at least 10 seconds” [7]. The peak incidence of OSAS in males is around 55 years of age, while in females it is around 65 years of age; before menopause, women report a lower prevalence of OSAS than men, whereas after this hormonal period, women become twice as numerous [8,9].

Despite the high number of patients with OSAS, this disorder is often underdiagnosed, while most patients with a certain diagnosis are poorly compliant in following the treatment process, such as the nocturnal application of continuous positive airways pressure (CPAP). CPAP is a non-pharmacological and non-surgical treatment and is considered the gold standard [10].

OSAS must be distinguished from central sleep apnea (CSA), which is caused by a functional alteration of the respiratory centers. To distinguish the two types of SDB and to avoid false positives, it is necessary to use, in addition to polysomnography, an electromyograph for the diaphragm muscle and, when possible, an air-filled balloon catheter to measure esophageal pressure [11].

The article presents a proposal for a new questionnaire to classify patients at risk for OSAS, taking into account associated comorbidities. It does not include questionnaire validation; thus, further studies will be needed to validate its use in the clinical setting. The use of the questionnaire is indicated for post-surgical patients and/or for patients who have not yet received a diagnosis of OSAS (and it could be used in outpatient clinical settings as well).

## Technical report

We designed the preventive questionnaire for OSAS (Pre-OSAS) based on 30 years of clinical experience, trying to identify and intercept potential patients with OSAS in advance, with the ultimate goal of improving the patient's clinical response. This is the first questionnaire clinicians and other professionals can use, accounting for comorbidities and sedentary lifestyles, as well as other factors already included in other evaluation approaches (Table 1).

**Table 1:** Pre-OSAS - the preventive questionnaire for obstructive sleep apnea syndrome The clinician puts a cross on the number corresponding to the presence of the disorder or pathology, while if the disorder or pathology is absent, no cross is put. At the end of the evaluation, the clinician must verify the sum of the scores obtained. * If one or more pathologies are present, the score is always 0.5. OSAS - obstructive sleep apnea syndrome

Items of the preventive questionnaire for OSAS: Pre-OSAS	Score
Snoring	1
Male sex >50 years	1
Female sex >65 years	1
Obesity body mass index >35 Kg/m^2 ^/ neck circumference ≥40cm	1
Diaphragmatic pathologies/dysfunctions (assessment with the Bordoni Diaphragmatic Test)	1
Pathologies/dysfunctions of the lingual complex (assessment with the Tongue Performance Test)	1
Daytime sleepiness	0.5
Cardiovascular diseases	1
Respiratory disorders	1
Previous thoracic surgeries	1
Diabetes/metabolic syndrome	1
Renal disorders	0.5
Liver pathologies	0.5
Depression/anxiety/stress	1
Cognitive decline	1
Neurodegenerative diseases	1
Tumors	1
Peripheral neuropathy	0.5
Otorhinolaryngological pathologies/disorders	1
Dysphagia	1
Ocular pathologies/disorders	0.5
Gastrointestinal pathologies/disorders	1
Dysphonia/chronic dry cough	1
Morphological craniofacial alterations	1
Alterations in taste/smell/hearing	1
Chronic undefined symptoms (dizziness, vitamin D deficiency, nocturia, slowed memory, involuntary movement of the lower limbs at night, dry mouth, facial pain, low back pain, neck pain, bruxism, functional alterations of the temporomandibular joint, headache, gout)*	0.5
Sedentary lifestyle	1
Pregnancy in the third trimester	1
Family history of OSAS	0.5
Total score	

The questionnaire consists of 29 items for assessment, with a maximum score of 25.5 (22 items with a score of 1 and seven items with a score of 0.5). In theory, as we have not yet validated the questionnaire, if the score from the assessment is greater than 13 (more than half of the total score), there is a high probability of upper respiratory tract dysfunction. The higher the score, the greater the probability that the patient may suffer from OSAS, and the clinician may decide to send the person for a more in-depth examination, such as polysomnography. It is a clinician-administered assessment and not a self-administered test. The clinician puts a cross on the number corresponding to the presence of the disorder or pathology (or circles the number or checks the box), while if the disorder or pathology is absent, no markings are made. At the end of the evaluation, the clinician must verify the total score. The pre-OSAS questionnaire can be used without requesting permission from the authors - the questionnaire is free to use.

We did not include the tongue position that can be discriminated by visual examination, such as the Friedman tongue position (also known as the modified Mallampati; see image in Appendix 1). The reason is that the shape and the anatomical space do not always necessarily coincide with the function. We preferred to include two active assessment items for the tongue (and the diaphragm) to better understand not only the tongue's function but also the respiratory system in general. In fact, breathing involves not only the tongue, but also the diaphragm [12].

For some assessment items, we have given a score of 1, while for others, we have given a score of 0.5. This depends on the weight that the literature gives to the problem/pathology present in the patient, where one is a score with a high percentage of occurrence in OSAS (for example, cardiovascular pathologies), while 0.5 is assigned for disorders or pathologies with a lower percentage of occurrence (for example, vertigo). The questionnaire is conducted during the patient's anamnesis by questioning the patient.

## Discussion

Clinical research is always evolving, and some dogmas related to OSAS evaluation are changing. For example, some authors place greater importance not on the value of AHI, but on the average duration of apneas/hypopneas [13].

It is not always possible to find a linear correlation between SDB/EDS and AHI values; the classification/severity of OSAS should not be based solely on AHI and daytime sleepiness symptoms, as clinical incongruities may result [4,5]. In most of the population evaluated and classified as OSAS patients, nocturnal sleepiness symptoms appear, regardless of AHI severity [5]. The clinician should also include the symptoms and related comorbidities** **[4]. About 80% of patients with OSAS present several comorbidities, which increase in percentage with the progression of the severity of the respiratory disorder [14,15].

Furthermore, patients classified with a mild disorder but symptomatic could be the most critical from the point of view of therapeutic management, and the use of CPAP could become a diagnostic and not therapeutic tool [4,8]. Nocturnal desaturation does not necessarily correlate with the presence of OSAS, but also with non-physiological structural alterations (dystrophy, severe scoliosis), as well as with visceral pathologies (chronic heart failure or chronic obstructive pulmonary disease), all of which do not involve upper airway obstruction. Furthermore, the interpretation of oximetry data for classifying patients with OSAS is not always linear [16].

Not all patients are the same and should be identified in more homogeneous phenotypes (clinical expression), which could be defined as: "A category of patients with OSA distinguished from others by a single or combination of disease features, in relation to clinically meaningful attributes (symptoms, response to therapy, health outcomes, quality of life)" [15]. The causative mechanisms (endotype) that determine the phenotype are always different and must be subjective: the phenotype and the endotype should allow a better choice of curative management path [14-16].

Knowing the presence of comorbidities allows us to identify subgroups of patients with OSAS and better manage treatment. For example, patients with moderate-severe obesity with the use of CPAP seem to suffer less cardiovascular damage, but only those who showed a greater number of comorbidities; or it seems that the effect of CPAP is more effective in males than in females [17].

Chronic hypoxia resulting from this nocturnal respiratory disorder generates a cascade of non-physiological metabolic events, such as oxidative stress, which cause an increase in the immune response with a systemic pro-inflammatory environment [18]. Systemic inflammation leads to structural alterations of the arterial and coronary vessels (endothelial dysfunction), chronic intervention of the sympathetic system, and a decrease in the parasympathetic system; an inability of the organism to manage blood pressure (hypertension, particularly nocturnal hypertension) and cardiac remodeling are recorded [7,18].

Another problem that is often underestimated is the dysfunction of the diaphragm muscle. A unilateral diaphragmatic lesion is often sub-clinical, and it is not easy to identify its presence; in this context, the OSAS symptoms become more severe [19]. If the diaphragm muscle does not contract correctly, it can cause OSAS [20]. Considering that the diaphragm is the main inspiratory muscle, it is governed by the respiratory centers of the upper airways; it would be useful to evaluate how this muscle behaves to identify patients with OSAS, even with the simple Bordoni Diaphragmatic Test (BDT) [12].

In conclusion, some risk factors can exacerbate the symptoms of OSAS or be a source of this disorder. The questionnaire we have proposed (to be validated) seeks to provide clinicians with a tool for a more careful and comprehensive evaluation of symptoms and disorders that can serve as a warning sign of OSAS (Figure 2).

**Figure 1:**
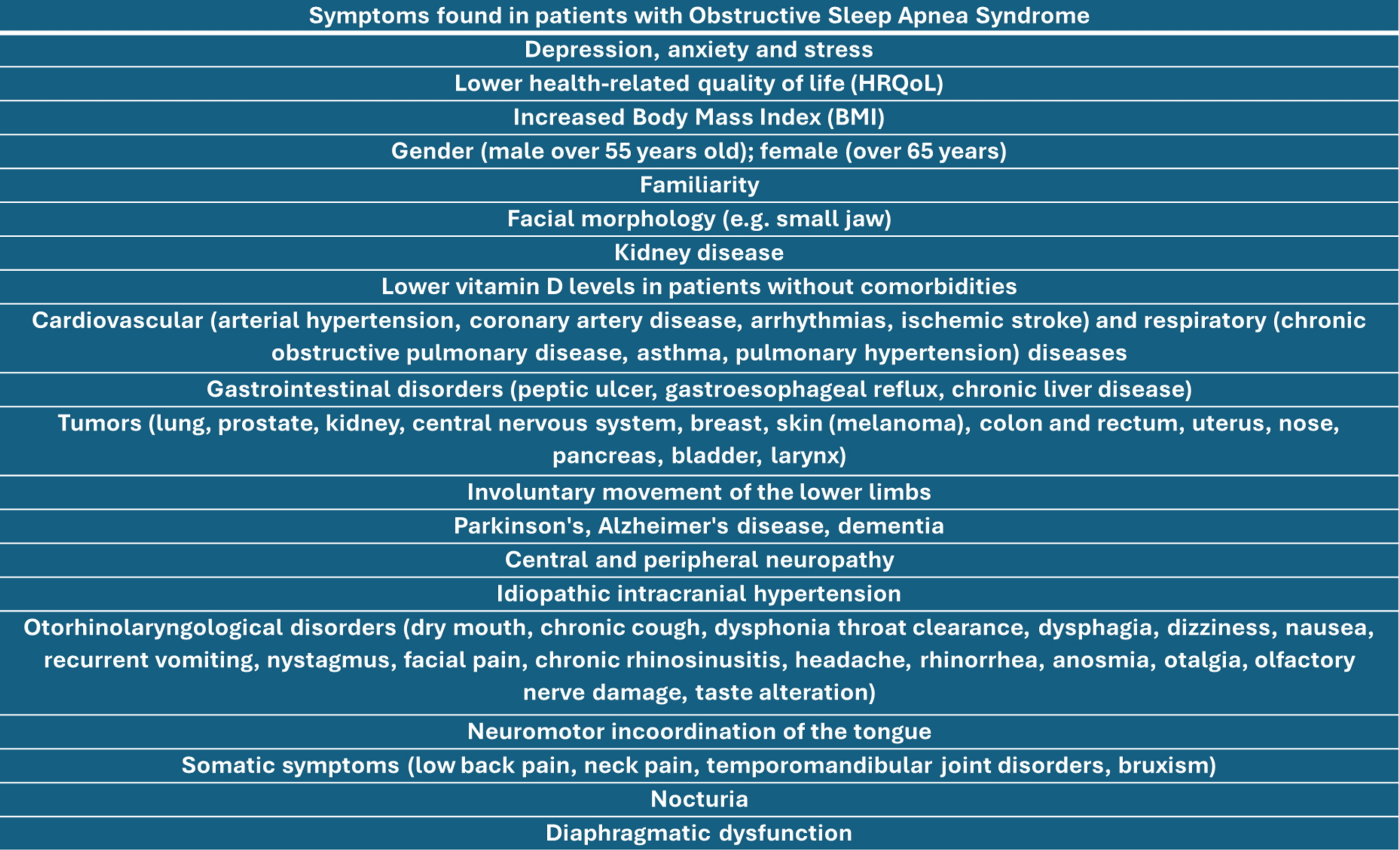
The concomitant pathologies or disorders that may be found in patients with obstructive sleep apnea syndrome Image created by Bruno Bordoni

Finally, we intend to publish a follow-up review article where we will compare our test and current tests. The test will have to undergo a validation process before being used in clinics.

## Conclusions

Obstructive sleep apnea syndrome (OSAS) is a disorder that affects approximately one billion people; this latter number is underestimated. OSAS is characterized by repeated apneas during the sleep cycle due to upper airway obstruction, caused by inadequate function and performance of the pharyngeal dilator muscle complex with respect to the negative intraluminal pressure forces resulting from inspiratory muscle contraction. Diagnosis is made based on the apnea-hypopnea index per hour from polysomnography. Not all questionnaires include the comorbidities of patients with OSAS, which are also common and can confuse the main problem of apnea. The article proposed a new questionnaire to preventively identify patients with OSAS, considering different comorbidity variables. The questionnaire presentation does not include validation, but further studies will be needed to validate its use in the clinical setting. The use of the questionnaire is more suitable for post-surgical patients and/or for patients who have not yet received a diagnosis of OSAS. The pre-OSAS questionnaire can be used without requesting permission from the authors - the questionnaire is free to use.

## Figures and Tables

**Figure 2:**
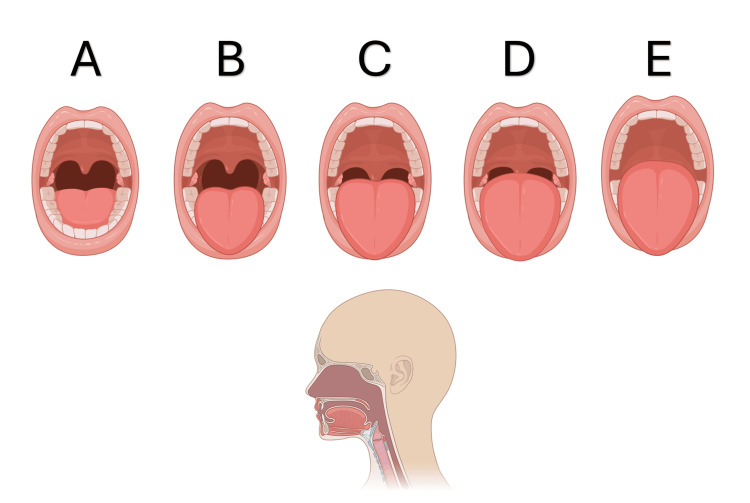
The four levels of tongue position The Mallampati score: B: the tonsils, uvula, and soft palate are completely visible; C: the hard and soft palate, the upper part of the tonsils, and the uvula are visible; D: the soft and hard palate, and the base of the uvula are visible; E: only the hard palate is visible. The higher the score, the greater the likelihood that the patient suffers from obstructive sleep apnea syndrome. Image A is a physiological tongue complex at rest, without tongue protrusion. The sagittal image below highlights the position of the tongue complex at rest. The image is the property of Bordoni Bruno, composed in Biorender.com
